# When Is Surgical Interference Justifiable in Cerebral Disease?

**Published:** 1898-05

**Authors:** Edward D. Fisher


### Selections and Abstracts.

### WHEN IS SURGICAL INTERFERENCE JUSTIFIABLE IN CEREBRAL DISEASE?

(*I. E.*, IN CEREBRAL GROWTHS, ABSCESS, EPILEPSY, MICROCEPHALUS, ETC.)*

By EDWARD D. FISHER, M. D.

The question of surgical interference in cerebral disease has been before the profession for some time. A few years ago, when it was found that with proper antiseptic precautions the brain and spinal cord could be handled with as little danger as the other organs of the body, it was supposed that a great field had been opened for cure in many cases hitherto regarded as hopeless. Indeed, many operations have been suggested simply on the ground that to do something, even if it was only the opening of the skull, might prove beneficial, so that in general paralysis, idiopathic epilepsy, etc., an operation has often been advised.

The pendulum has swung in the other direction at present. As an operation had often been done in inappropriate cases, with little if any result, and as also death was not infrequent either from inefficient methods or from unskilled operators, all operation was deplored. There has always been a true middle course to pursue. In fact, in certain cases a physician is culpable who does not advise surgical interference, even although no positive promise can be made of curative results, and even when only relief can be hoped for. It must always, indeed, be remembered that these operations are capital, and that therefore danger to life is always present. No inexperienced surgeon should undertake them, without at least careful study of the methods and indications. Another side is always to be remembered, and that is, that life is often prolonged or made more endurable by operation, and this is important enough to take into consideration. I am glad that the conservative view of this operation has been generally accepted, and that men do not so often rush in where angels fear to tread as was formerly the case.

Operations, therefore, for general paralysis, a disease whose pathology shows it to be a widespread inflammation of the membranes and cortex of the brain, should not be undertaken. There is no basis for operation in these cases.

I would not have it understood that I am an earnest advocate for surgical interference in cerebral cases, but this much I would say, that knowing now that the brain can be handled (indeed with caution) without injury to its substance, we

*Read before the Society of Alumni of Bellevue Hospital, January 5, 1898.

should no more hesitate to open into it than we should to open up the abdominal cavity; indeed, there is usually less shock in these cases than in abdominal cases. A certain number of cases, therefore, urgently demand, all other conditions being favorable, immediate operation—such as depression of the skull from fracture; meningeal hæmorrhage, especially traumatic, but not necessarily only these cases. Rarely if ever does intracerebral hæmorrhage indicate it, for from the very condition of things it means that the brain substance itself has been destroyed, and the removal of the blood could not restore the destroyed cerebral substance, and, again, the situation of the blood in the region of the internal capsule is too deeply placed to warrant removal.

The cause of the lack of success in these operations lies mostly in the fact that we are dealing usually with incurable conditions or irremovable complications. For instance, tumors of the brain are only "operable" in a small percentage of cases, say ten per cent., and out of this small number only a possible ten per cent. can be relieved. The explanation of this is, that the growth is often situated so deeply that its removal would cause such extensive ablation of the brain as to cause death; or, again, it is so situated, as at the base of the brain, that it can not be reached.

Accepting all these difficulties, there are certain strong indications for operation. One case which has been saved by operation demands that each case of that nature should have like opportunities of relief. The same may be said of localized epileptic seizures—whether traumatic in origin or not. The knowledge of cerebral topography is so accurate to-day that at least in these cases we know where to look for the lesion, and if one case can be recorded as benefited, although it is known that the majority do not prove successful, it is our duty to operate, provided other means have failed to bring relief.

Some of the special indications for operation are the following, therefore: 1. Fracture of the skull, causing compression with resulting paralysis, epileptic seizures, or coma. This would in no case be objected to, and was the practice long before the days of so-called cerebral surgery. 2. Meningeal hæmorrhages, traumatic or occurring in pachymeningitis hæmorrhagica. 3. Tumors of the brain when situated near the cortex of the brain or even in the cerebellum, but not when deeply situated or at the base. This last statement I would modify by saying that when the tumor is not thought to be a removable one a partial operation may be indicated, as the removal of a large area of the skull often relieves certain marked symptoms of tumor, as vomiting, headache, and convulsions. I have seen beneficial results of that nature in a number of cases in which that was all that could be attempted. 4. Localized epileptic seizures of the so-called Jacksonian type. I would include in this class cases, whether due to injury or arising from unknown causes—that is, so-called idiopathic epilepsy—if limited to special parts of the body, as the arm, leg, or face, or all three if only one side of the body is involved. In such cases I would advise the excision of these cerebral centers. This, indeed, results in paralysis, perhaps a permanent form; but in many of these patients we have already a certain degree of paralysis, and in that case we simply increase a previous disability. 5. The last indication which I shall mention for surgical interference is cerebral abscess, and especially in the form most commonly presented to us—that following otitis media. I will not include under this head operations in microcephalia or in infantile cerebral hemiplegia with epilepsy, although in some cases, owing to the otherwise hopeless character of these conditions, I am in favor of operative interference. It is too large a subject to take up on this occasion.

In conclusion, while not wishing to describe the methods of operation, I would urge that in cerebral operations a large area of the skull be removed. It both enables us to examine the brain better when exposed, and also, if benefit is to be obtained from relief of cerebral pressure, it surely increases that chance, and also it scarcely increases the danger of the operation. The removal of a mere button of bone with the trephine certainly exposes the patient to some danger, and rarely accomplishes much otherwise.

I will now relate in brief the history of a few cases, successful and otherwise, which have been under my care:

A. B., a boy, aged fourteen years, gave a history of a fall from a tree, injuring right side of head, causing some depression of skull. Five years later epileptic attacks ensued, for which a button of bone, somewhat anterior to the motor areas, had been removed without benefit. A large area of bone over the motor area was removed by Dr. George Woolsey. On the lower surface a spiculum of bone was found, which had extended into the hand center. This was removed and the bone flap replaced, perfect union resulting. This boy was kept under observation and bromide administered. The attacks became less frequent, and when last heard from, three years following the operation, no seizures had taken place for a year, and the bromides had been long discontinued.

A second case was that of a Greek who came under my observation at the University College Dispensary. He gave the history of a blow on the left side of the head. This was followed by a localized convulsion of the right side of the body, commencing with a sensory disturbance—*i. e.*, tingling in the tongue and lips and fingers of that side.

This patient also had had a button of bone removed previous to coming under my care.

Dr. Woolsey removed a large area of the skull over the motor area by the bone-flap operation.

The dura at the site of the previous operation was found thickened and adherent, and was therefore removed. The bone was replaced. The patient was discharged improved and returned to his occupation in a circus. He returned some months later to the hospital and a second operation was performed, removing again a thickened membrane which was adherent to the skull. Again improvement followed for a time only.

A third and a fourth operation was performed; in the last one the bone was not replaced, but a cap of celluloid was substituted. The result has finally on the whole been favorable, as the attacks are very infrequent and not severe, the patient being able to carry on his occupation.

There may be occasion for further surgical interference. Apparently no bad results from shock follow the operation. A full report of this case will be made at a later date.

The following case I shall merely refer to as showing how an extensive growth may present very few symptoms, and as interesting also in that apparently the shock of the operation, possibly owing to the size of the tumor, resulted fatally. I will also pass the specimen around.

A. B., laborer, about forty years old, was seen at the clinic of the University Medical College for the first time. He complained only of a slight headache, and said he had convulsions, after which he had some weakness in his hand, and dragged his leg in walking. He said he had been in the Homeopathic Hospital for some months, and there a button of bone had been removed from the skull. He was sent to Bellevue Hospital for observation. Examination showed slight paresis of the right hand and considerable ataxia and exaggerated knee-jerk on the same side. The patient was around the wards for some weeks, and in that time only one convulsion was reported. The eyes on examination gave no evidence of optic neuritis. The headache was never severe.

The operation was badly borne, the pulse from the first being weak.

The skull was removed over the motor area and the large growth became evident. It would have been impossible to remove it entire.

The patient within a few hours succumbed.

This patient showed very few symptoms then or at any time. The absence of paralysis can only be explained by a gradual pressing aside of the fibers, and their thus escaping destruction.

[The author then presented two other tumors and gave brief histories of the cases. No operation was performed, and none was practicable.]—*New York Medical Journal.*

